# Collagen Supplementation on Tendon-Related Structural and Performance Outcomes: A Systematic Review

**DOI:** 10.3390/jfmk11010130

**Published:** 2026-03-23

**Authors:** Albert Buchalski, Michael Jeanfavre, Colby Altorelli, Gretchen Leff

**Affiliations:** Stanford Health Care and Outpatient Orthopedic, Sports Medicine Rehabilitation Department, Redwood City, CA 94063, USA

**Keywords:** connective tissue remodeling, hydrolyzed collagen, resistance training, tendon stiffness, rate of force development, musculoskeletal adaptation

## Abstract

**Background:** Tendons adapt to mechanical loading by increasing cross-sectional area (CSA), stiffness, and matrix organization, with structural remodeling critical for both rehabilitation and performance. Collagen supplementation has been proposed to enhance this process by supplying key amino acids for collagen synthesis; however, inconsistent results across trials have limited its clinical and athletic application. **Methods:** A systematic review of randomized controlled trials evaluating collagen supplementation in humans was conducted. PubMed, EMBASE, CINAHL, and Web of Science were searched from database inception through May 2025. Risk of bias was assessed using the PEDro scale (≥6/10 classified as good-to-excellent quality). Due to substantial heterogeneity in supplementation protocols, training modalities, and outcome measures, results were synthesized narratively without meta-analysis. Data extraction included collagen type, dose, training modality, intervention duration, and outcome measures. **Results:** Of 887 unique citations, eight RCTs (*n* = 257; ages 18–52; 246 M:11 F) met the inclusion criteria. All studies incorporated resistance or plyometric training (3–15 weeks). Three of four studies reported significantly greater increases in tendon CSA in collagen groups versus placebo. Four studies investigated tendon stiffness and Young’s modulus; the two using higher doses (15–30 g/day) demonstrated significant between-group improvements favoring collagen, while lower-dose studies (~5 g) showed only within-group effects. Muscle strength improved with training in all trials, but no additive effects of collagen were observed. One study reported improvements in eccentric rate of force development and deceleration impulse with collagen, though gross explosive metrics (e.g., jump height) were unaffected. **Conclusions:** Collagen supplementation (15–30 g) with vitamin C (≥50 mg) may enhance tendon remodeling when combined with high-intensity resistance training (≥70% 1 RM). The current literature suggests strong evidence (GRADE A) for increases in tendon CSA and stiffness, strong evidence (GRADE A) against an effect on muscle strength, and conflicting evidence (GRADE C) for muscle cross-sectional area and physical performance. Limitations include small sample sizes, heterogeneous protocols, and short intervention durations.

## 1. Introduction

Tendons are essential connective tissues that transmit muscular forces to bone, facilitating movement, joint stability, and elastic energy storage during dynamic tasks [1,2]. Predominantly composed of Type I collagen, tendons adapt structurally and functionally to mechanical loading through increases in cross-sectional area (CSA), stiffness, and extracellular matrix (ECM) organization, which collectively enhance force transmission efficiency and injury resilience [3]. These adaptations occur primarily through tenocyte activation and ECM remodeling mediated by strain-induced signaling pathways involving growth factors and mechanotransduction processes [3,4].

Collagen supplementation has emerged as a promising adjunct to mechanical loading for promoting tendon remodeling [5,6,7]. Hydrolyzed collagen and collagen peptides contain high concentrations of glycine, proline, hydroxyproline, and hydroxylysine, amino acids critical as biochemical precursors to collagen synthesis [6,8,9]. When co-ingested with vitamin C, an essential cofactor required for proline and lysine hydroxylation and subsequent collagen crosslinking, these amino acids reach peak serum concentrations approximately 60 min post-ingestion, ensuring their availability during the period of enhanced collagen synthesis stimulated by mechanical loading [8,9]. This synergy suggests a potential optimization of exercise-driven remodeling through strategic nutrient timing.

Tendon remodeling is evaluated by biomechanical properties, including cross-sectional area (CSA), stiffness, and Young’s modulus, each reflecting distinct aspects of tendon quality and function [10,11,12]. CSA typically increases as an adaptive response to mechanical overload; however, enlarged CSA in tendinopathy often reflects pathological changes such as collagen disorganization, neovascularization, and extracellular matrix swelling, resulting in mechanically impaired tendons [13,14]. Tendon stiffness, defined as resistance to elongation under load, directly influences force transmission efficiency and performance metrics, including rate of force development and reactive strength [2,12]. Young’s modulus, calculated as stress divided by strain, normalizes stiffness relative to tendon size, isolating intrinsic tissue quality independently of tendon dimensions [15,16]. Clinically, this distinction is crucial, as tendinopathic tendons commonly demonstrate increased CSA alongside reduced stiffness and modulus, indicating compromised mechanical integrity [17,18]. Rehabilitation protocols aim to restore not only tendon size but also tissue quality and mechanical efficiency, while athletic populations may further benefit from increased stiffness and modulus to optimize energy storage and neuromuscular efficiency during dynamic stretch-shortening movements [2,19].

Although collagen supplementation has traditionally been investigated for its effects on joint pain and cartilage health [5,7], there is emerging research toward its potential role in enhancing tendon and muscle adaptation when combined with resistance training. Studies consistently demonstrate that collagen ingestion, when timed around mechanical loading, can stimulate collagen synthesis and may improve tissue mechanical properties [9,20]. However, findings from trials remain mixed: while some report improvements in tendon CSA and stiffness, others show minimal effects on muscle strength, hypertrophy, or performance [6,7]. This inconsistency highlights the lack of clearly defined evidence-based guidelines regarding optimal collagen supplementation protocols in both clinical rehabilitation and athletic performance settings.

Collagen supplementation is particularly relevant in populations exposed to high levels of tendon loading, such as athletes participating in running, jumping, and field-based sports, where repetitive mechanical strain imposes substantial demands on tendon structure, function, and load-transfer capacity [1,2,3,4]. From a clinical perspective, tendon remodeling is also central to rehabilitation, as pathological presentations may involve increased cross-sectional area accompanied by compromised mechanical integrity, including reductions in stiffness and material properties [13,14,17,18]. Accordingly, interventions that support extracellular matrix remodeling may have translational relevance across both rehabilitation and performance contexts, particularly given the established functional importance of tendon stiffness and material properties for efficient force transmission and stretch-shortening cycle performance [2,19].

The purpose of this systematic review is to critically evaluate the effects of collagen supplementation, alone or in combination with vitamin C, on tendon-related structural and performance outcomes, including tendon CSA, stiffness, Young’s modulus, muscle strength, muscle CSA, and physical performance. By synthesizing the available evidence, this review aims to clarify optimal dosage, timing, and implementation strategies to inform evidence-based application in clinical rehabilitation and athletic performance settings.

## 2. Methods

This systematic review was conducted in accordance with the Preferred Reporting Items for Systematic Reviews and Meta-Analyses (PRISMA) guidelines [15,16,21]. This systematic review was prospectively registered with the International Platform of Registered Systematic Review and Meta-Analysis Protocols (INPLASY; Registration No. INPLASY2025110059). The full protocol is publicly accessible through the INPLASY registry. No amendments were made to the registered protocol.

### 2.1. Study Identification and Search Strategy

**Figure 1:**
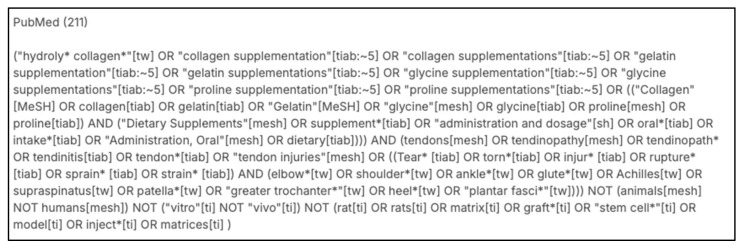
PubMed search strategy.

**Figure A2:**
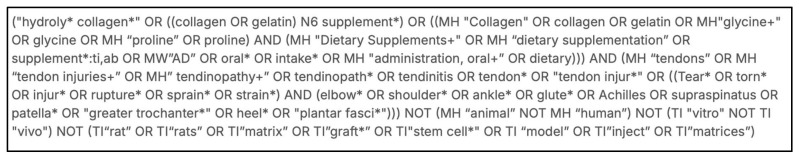
CINAHL search strategy.

**Figure A3:**
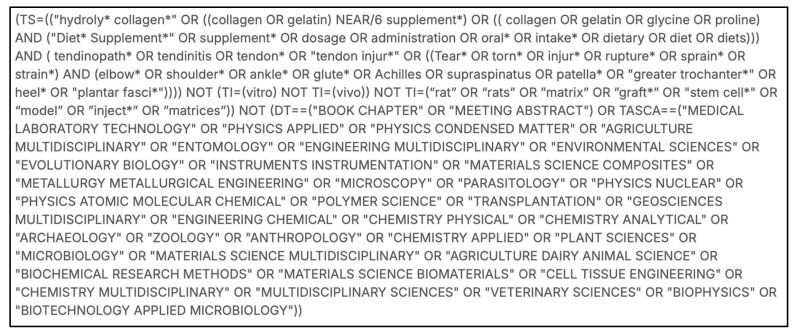
Web of Science search strategy.

A comprehensive literature search was performed across PubMed, EMBASE, CINAHL, and Web of Science databases in May 2025. The search was limited to studies published in English. The search strategy was developed in collaboration with a medical school librarian, including the accurate use of Boolean modifiers and standardized translation of search terms across databases, in accordance with the predefined research question and inclusion criteria. The final search strategy for PubMed and the respective results are shown in Figure 1. Search strategies for EMBASE, CINAHL, and Web of Science are presented in Appendix A, Figure A2, Figure A3.

Additionally, to ensure a comprehensive identification process, hand-selected articles that were identified through the study selection process and by scouring the references of the included articles were also included.

### 2.2. Eligibility Criteria

The research question guiding this systematic review was developed using the PICO framework, as recommended by the PRISMA guidelines [22]. The PICO question variables, study elements, and respective inclusion and exclusion criteria are outlined in Table 1. Although several study designs were initially eligible according to the predefined inclusion criteria, only randomized controlled trials met the final inclusion criteria following the screening process.

### 2.3. Study Selection

The initial search results of the different databases were combined, duplicates were deleted, and the resulting studies were filtered independently according to the specified inclusion and exclusion criteria by two members of the research team (Author 1, A.B.; and Author 2, C.A.) using a citation manager, Zotero 7 (Corporation of Digital Scholarship), and systematic review software management system, Covidence (Veritas Health Innovation, Melbourne, Australia). Discrepancies in the filtering of the search results were discussed by the two independent reviewers (Author 1, A.B.; and Author 2, C.A.).

### 2.4. Data Extraction

Data elements of identified full-text articles were prospectively determined based on the predefined PICO (Population, Intervention, Comparator, and Outcome) question and the purpose of the current study. The population included human participants of any age, sex, or ethnicity with either healthy tendons or clinically diagnosed tendon pathology. The intervention was collagen supplementation, including hydrolyzed collagen, gelatin, or collagen peptides, with or without additional amino acids such as proline or glycine. The comparator was a control group receiving a placebo, no treatment, or a different form of collagen supplementation. The outcomes were tendon health-related measures, including tendon cross-sectional area, tendon mechanical properties, muscle performance, physical performance, and pain levels associated with tendon injuries. Effect estimates were extracted as reported in the original trials, including between-group mean differences, within-group changes, and associated *p*-values or confidence intervals when available. Due to methodological heterogeneity in collagen formulation, dosage, intervention duration, and outcome measurement techniques, a quantitative meta-analysis was not performed. Studies were grouped for synthesis according to predefined outcome domains, including tendon structural outcomes, muscle structural outcomes, strength measures, and functional performance outcomes. Only studies reporting comparable outcome measures were synthesized together narratively within each domain. When change-score variability was not directly reported, it was estimated from available summary statistics where possible; no additional imputation or statistical transformation procedures were performed. No formal subgroup analyses or meta-regression procedures were conducted to explore sources of heterogeneity. No sensitivity analyses were conducted. Due to the absence of quantitative meta-analysis, formal assessment of publication bias or small-study effects was not conducted.

### 2.5. Summary of Measures and Synthesis of Results

The results were synthesized into structured graphs summarizing primary outcome domains across studies, including tendon structure, mechanical properties, muscle performance, and physical performance. Findings were compared based on variations in collagen type, dosage, ingestion timing, frequency, and the nature of concurrent exercise interventions.

Based on the trends observed, a proposed evidence-informed protocol for collagen supplementation in tendon health is presented, integrating both research findings and clinical reasoning to support practical implementation in athletic and rehabilitative settings.

### 2.6. Risk of Bias Assessment

The risk of bias and methodological quality of all included RCTs were assessed using the Physiotherapy Evidence Database (PEDro) scale. Two independent reviewers (Author 1, A.B.; and Author 2, C.A.) completed the assessments. Any discrepancies between reviewers were resolved through discussion to reach consensus. The PEDro scale evaluates criteria related to internal validity and has sufficient statistical information to guide clinical decision-making through scoring 11 items, with the total score ranging from 0 to 10, as the first item (external validity) is not included in the final score.

The level of evidence and grade of recommendation for all included RCTs were assessed using the PEDro (Physiotherapy Evidence Database) scale, a validated appraisal tool used in physical therapy and rehabilitation research [23]. The PEDro scale includes 11 criteria, 10 of which contribute to the total score, with items scored as either present (1) or absent (0). Based on established thresholds, scores of ≤3 indicate poor quality; 4–5, fair quality; 6–8, good quality; and 9–10, excellent quality.

### 2.7. Level of Evidence and Recommendation

The level of evidence for each included reference was assessed using the Oxford Centre for Evidence-Based Medicine (OCEBM; Oxford, UK) criteria (Table 2). The OCEBM framework, first introduced in 1998 and updated in 2011, ranks evidence from Level I, representing the highest quality, to Level V, representing the lowest quality, based on study design, use of randomization and blinding, and the degree of potential bias [24].

The overall grade of recommendation for collagen supplementation was determined using the Grading of Recommendations Assessment, Development, and Evaluation (GRADE) approach (Table 3). Developed in 2000, the GRADE system applies an alphabetical scale, from A, representing the strongest recommendation, to F, representing the weakest recommendation. This approach incorporates both the quality of evidence and the strength of the recommendation to facilitate the application of research findings to clinical decision-making [25].

The use of both OCEBM and GRADE was selected because these systems are endorsed by the American Physical Therapy Association (APTA) for grading the quality of evidence in Clinical Practice Guidelines [27].

The GRADE framework was applied to provide a structured evaluation of the strength of evidence derived from the included randomized controlled trials. Because all included studies were randomized designs demonstrating good-to-excellent methodological quality based on PEDro scoring, the grading of recommendations reflects the predominance of higher-level evidence rather than pooled quantitative effect estimates. Given the heterogeneity in supplementation protocols, training interventions, and outcome measures across studies, the GRADE classifications should be interpreted as a structured summary of the available evidence rather than definitive clinical guideline recommendations.

## 3. Results

### 3.1. Study Selection

**Figure 2:**
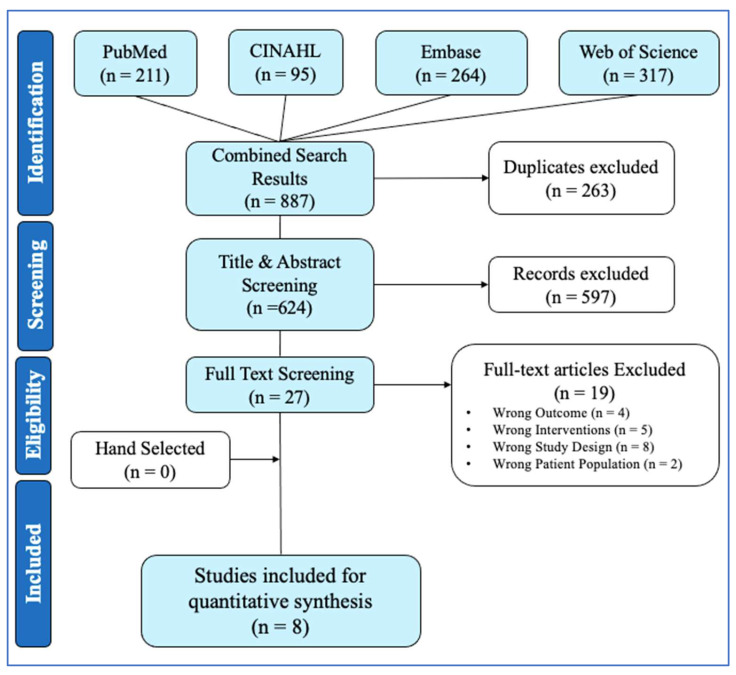
The PRISMA flow diagram.

A total of 624 articles were identified by the initial search results after the removal of duplicates. Of the 27 articles read in full, eight articles were deemed appropriate for final analysis. All eight articles included were RCTs. Figure 2 outlines the study selection process in a PRISMA flow diagram. A summary of the outcome characteristics is provided in Appendix B.

### 3.2. Study Characteristics

Study characteristics extracted from each article included the primary author, year of publication, participant demographics, study design, methodological details, and primary outcomes measured. Additionally, each study’s primary results were documented to facilitate comparison across intervention types and outcome domains. These characteristics are summarized in Table 4.

### 3.3. Risk of Bias Assessment

**Figure 3:**
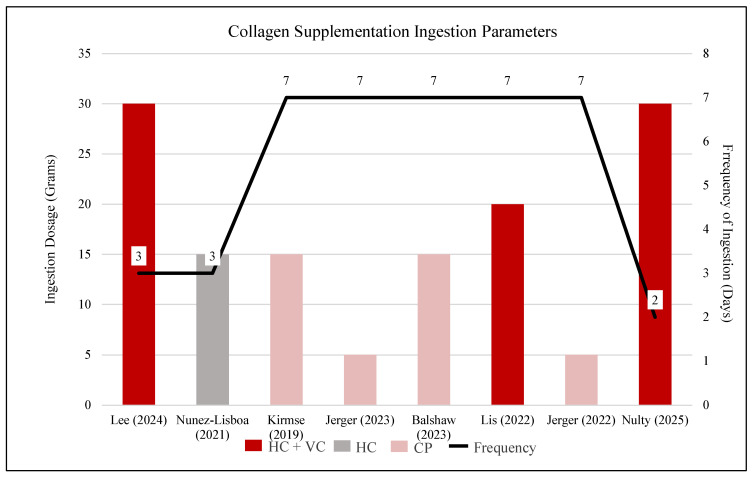
Collagen supplementation ingestion parameters. Note: HC, hydrolyzed collagen; VC, vitamin C; CP, collagen peptide [28,29,30,31,32,33,34,35].

The RoB assessment results for the RCTs are summarized in Table 5. The RCTs in this review received PEDro scores ranging from 7 to 9, reflecting good-to-excellent methodological quality. The highest risk of bias was Jerger et al. [34], Lis et al. [33], and Nunez-Lisboa et al. [29]. The lack of blinding in rehabilitation and physical therapy research is well documented, and the risk-of-bias assessments in this review further corroborate this limitation [36]. However, the RCTs were deemed to have at least “good” quality, as seen in Figure 3. Individual randomized controlled trials are classified as Level 2 evidence, supporting the methodological strength of the included studies [24].

### 3.4. Statistical Synthesis

No quantitative meta-analysis was performed due to substantial heterogeneity in collagen formulation, dosage, intervention duration, co-interventions, and outcome measurement methods across the included trials. Accordingly, no pooled effect estimates or statistical heterogeneity measures were calculated. No formal investigations of heterogeneity were conducted. No sensitivity analyses were conducted. Reporting bias was not formally assessed, because no quantitative synthesis was performed.

### 3.5. Collagen Ingestion

Collagen supplementation protocols varied in both dosage, frequency, and type among the eight included studies. Two of the studies [28,35] employed higher doses of 30 g, one study [33] used a dose of 20 g, three studies [29,30,32] administered 15 g, and two studies [31,34] used five grams. Ingestion frequency also differed, with five studies administering collagen daily (7 days/week), two studies following a 3-day/week protocol [28,29], and one study [35] implementing a twice-weekly regimen. Three types of collagen were observed: hydrolyzed collagen (HC), collagen peptides (CP), and a combination of hydrolyzed collagen with vitamin C (HC + VC). A visual summary of ingestion dosage, frequency, and collagen type across all included studies is presented in Figure 3.

### 3.6. Training Protocol

**Figure 4:**
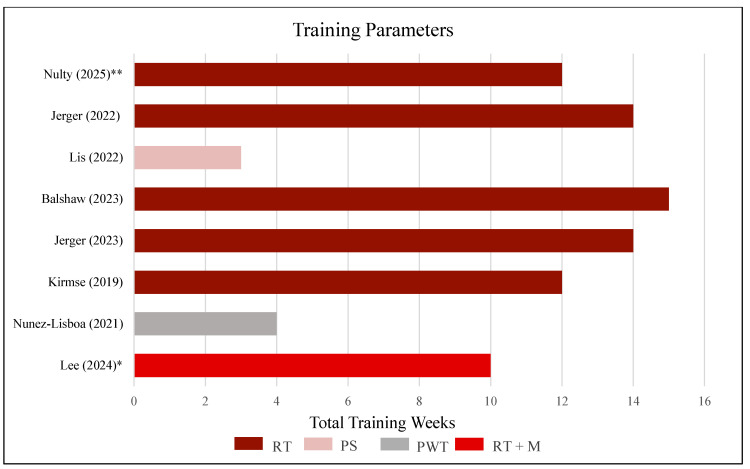
Exercise intervention frequency and training modes across included studies. Note: RT, resistance training; PS, plyometric-specific exercise; PWT, power training; RT + M, resistance training + match. Asterisks denote training frequency: ** indicates two training days per week, * indicates five training day per week, and the absence of an asterisk represents three training days per week [28,29,30,31,32,33,34,35].

Training interventions varied in duration, type, and weekly frequency among the eight included studies. Five studies [30,31,32,34,35] utilized traditional resistance training (RT) protocols, with durations ranging from 12 to 15 weeks. One study [28] combined resistance training and a sports-specific match day (RT + M) for 10 weeks, while another [29] employed a power-training (PWT) protocol over 4 weeks. A plyometric-specific (PS) intervention for 4 weeks was used by Lis et al. [8]. Training frequency varied, with five studies utilizing a 3-day/week schedule, one study [35] implementing 2 days/week, and one study [28] utilizing a 5-day/week protocol. A detailed visual comparison of training type, total training weeks, and frequency is provided in Figure 4.

### 3.7. Tendon Cross-Sectional Area (CSA)

**Figure 5:**
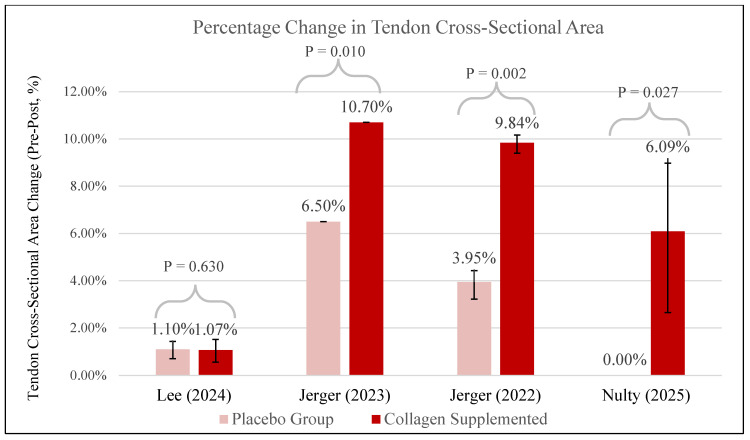
Change in tendon cross-sectional area [28,31,34,35].

Four studies evaluated the effect of collagen supplementation on tendon CSA. Studies by Jerger [31,34] and Nulty [35] demonstrated increases in CSA favoring the collagen-supplemented groups. In Jerger [34], a 14-week resistance training program combined with specific collagen peptides led to a 9.84% increase in Achilles tendon CSA, compared to 3.95% in the placebo group (*p* = 0.002). Similarly, Jerger [31] found a 10.7% increase in patellar tendon CSA in the collagen group versus 6.5% in placebo (*p* = 0.010), with significant effects observed at multiple tendon regions. Nulty [35] implemented a 12-week lower-extremity resistance-training program combined with 30 g of hydrolyzed collagen and 50 mg of vitamin C, which resulted in a 6.09% increase in patellar tendon CSA, compared to no change in the placebo group (*p* = 0.027). Conversely, Lee [28] reported no significant difference between groups in tendon CSA following training, with changes of 1.07% in the collagen group and 1.10% in the placebo group (*p* = 0.630). A summary of pre-to-post CSA percentage changes across studies is presented in Figure 5, highlighting the variability in tendon remodeling responses to collagen supplementation.

### 3.8. Tendon Stiffness

**Figure 6:**
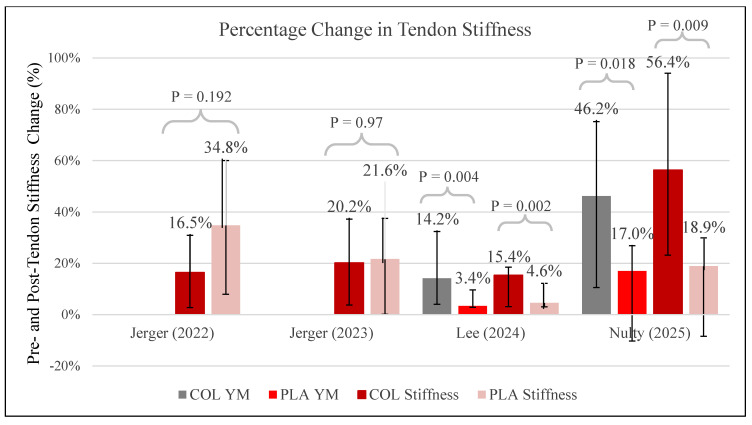
The percentage of tendon stiffness change pre- and post-intervention. Note: COL, collagen; PLA, placebo; YM, Young’s modulus [28,31,34,35].

Four studies [28,31,34,35] evaluated the effect of collagen supplementation on tendon stiffness. Lee [28] reported significantly greater percentage increases in tendon stiffness and Young’s modulus in the collagen group (+15.4 ± 3.1% stiffness; +14.2 ± 4.0% YM) compared to placebo (+4.6 ± 3.0% stiffness; +3.4 ± 2.8% YM) (*p* < 0.001). Nulty [35] observed stiffness increases of +661 ± 331 N/mm (+56.4%) in the collagen group and +247 ± 305 N/mm (+18.9%) in the placebo group (*p* = 0.009, d = 2.0). Young’s modulus also increased more in the collagen group (+0.21 ± 0.13 GPa, d = 1.83) compared to placebo (+0.09 ± 0.13 GPa, d = 0.662), with a significant group–time interaction (*p* = 0.018). Jerger [34] reported increases in Achilles tendon stiffness from 389.5 ± 135.0 N/mm to 453.7 ± 158.8 N/mm (+16.5%) in the collagen group and from 401.5 ± 102.6 N/mm to 541.1 ± 132.5 N/mm (+34.7%) in the placebo group, with significant within-group effects but no interaction effects (*p* = 0.192). Similarly, Jerger [31] found that patellar tendon stiffness increased from 1422.0 ± 336.9 N/mm to 1708.9 ± 410.2 N/mm (+20.2%) in the collagen group and from 1459.3 ± 501.3 N/mm to 1774.9 ± 554.6 N/mm (+21.6%) in placebo, again with no significant group × time interaction (*p* = 0.97). A summary of pre-to-post percentage changes in stiffness and Young’s modulus across studies is presented in Figure 6.

### 3.9. Physical Performance

Lis et al. [33] and Nulty et al. [35] both examined the effects of collagen supplementation on performance. Lis et al. [33] found that both groups increased maximal isometric squat force (PLA: +7.09 ± 2.80%; HC + C: +7.81 ± 2.60%), but only the HC + C group maintained rate of force development (RFD) levels from baseline (−2.13 ± 5.20%), compared to a decrease in the PLA group (−16.20 ± 4.00%). A significant group × time interaction was reported for RFD (*p* = 0.04, d = 0.5), which was reduced to *p* = 0.07 when an outlier was removed. The HC + C group also showed greater eccentric RFD (*p* = 0.04, d = 0.6) and eccentric deceleration impulse (*p* = 0.03), with improvements in eccentric deceleration RFD (*p* = 0.008; d = 0.5). No between-group differences were observed for countermovement jump (CMJ) height, squat jump (SJ) performance, or reactive strength index (RSI). Nulty [35] reported that both groups improved absolute RTD (*p* = 0.022,) and normalized RTD (*p* = 0.020) following resistance training, but no significant group × time interaction was found for RTD (*p* = 0.712), peak RTD (*p* = 0.637), or explosive torque at any time point (50, 100, or 150 ms; all *p* > 0.05). No between-group differences were observed for CMJ height and power, or broad jump distance.

### 3.10. Muscle Volume/Cross-Sectional Area

Among the four studies [30,32,34,35] investigating the effects of collagen supplementation on muscle cross-sectional area (CSA) or thickness, two demonstrated statistically significant benefits of collagen over placebo, while two did not. Jerger [34] reported a significant group × time interaction (*p* = 0.014), indicating greater improvements in gastrocnemius muscle thickness in the collagen peptide (SCP) group (2.18 ± 0.24 cm to 2.34 ± 0.22 cm) compared to the placebo group (2.15 ± 0.42 cm to 2.20 ± 0.39 cm). Similarly, Balshaw [32] found significantly greater increases in the collagen group for quadriceps volume (+15.2% vs. +10.3%; *p* = 0.032) and total trained muscle volume (+15.7% vs. +11.4%; *p* = 0.026). A significant group × time interaction was also observed for vastus medialis volume, which showed a 61% greater relative increase in the collagen group (+15.6% vs. +9.7%). In contrast, Kirmse [30] reported significant main effects of time (*p* < 0.05) for muscle thickness, leg circumference, and type II muscle fiber CSA, but no significant group × time interactions (*p* > 0.05), suggesting similar improvements across groups. Nulty [35] found no additional benefit of hydrolyzed collagen supplementation on muscle thickness, with a non-significant group × time interaction (*p* = 0.714), indicating that resistance training alone accounted for the observed hypertrophy.

### 3.11. Muscle Strength

Across all six included studies [30,31,32,33,34,35], resistance training significantly improved muscle strength over time (*p* < 0.05); however, no statistically significant group x time interactions were observed in any trial. Collagen supplementation did not confer additional strength benefits beyond resistance training alone. These findings were consistent across multiple strength measures and muscle groups, reinforcing that collagen’s effects on muscular strength are negligible in individuals when compared to placebo supplementation. Additional outcome details, including test-specific comparisons, are presented in Table 6.

**Table 6:** Muscle strength within-group effects.

Author (Year)	Muscle Strength Tests	Group × Time Interaction	Between-Group Interpretation
Kirmse [30]	SL Ext. MViC, SQ 1 RM, DL 1 RM, BP 1 RM, BOR 1 RM	No sig. (*p* = 0.477–0.768), SQ trend *p* = 0.054	No between-group difference
Jerger [34]	PF MVT	No sig. (*p* = 0.629)	No between-group difference
Balshaw [32]	KE MViC, KE 1 RM, KF MViC, Absolute Torque, Torque expressed relative to MVT (torque at 50 ms intervals)	No sig. (*p* = 0.703–0.929 for %Δ; *p* = 0.054–0.862 for absolute)	No between-group difference
Lis [33]	Maximal isometric SQ	No sig. (*p* = 0.32)	No between-group difference
Jerger [31]	LP 1 RM, KE 1 RM	No sig. (*p* = 0.396, 0.805)	No between-group difference
Nulty [35]	KE MViC, LP 10 RM	No sig. (*p* > 0.05)	No between-group difference

Note: 1 RM, one repetition maximum; 10 RM, ten repetition maximum; BOR, bent-over row; BP, bench press; DL, deadlift; Iso. Squat, isometric squat; KE, knee extension; KF, knee flexion; LP, leg press; MViC, maximal voluntary isometric contraction; MVT, maximal voluntary torque; PF, plantarflexion; RTD, rate of torque development; SL Ext, single leg extension.

### 3.12. GRADE of Recommendations

According to the Grading of Recommendations Assessment, Development, and Evaluation (GRADE) Working Group Criteria, collagen supplementation suggests a Grade A recommendation for increasing tendon cross-sectional area and tendon stiffness. This rating is based on eight randomized controlled trials, all of which were rated as good or excellent methodological quality on the PEDro scale, with consistent findings supporting these outcomes. There is a Grade A recommendation against an effect on muscle strength, and the evidence for muscle cross-sectional area and physical performance is GRADE C.

## 4. Discussion

The purpose of this systematic review was to evaluate the effects of collagen supplementation on tendon-related outcomes, including tendon CSA, tendon stiffness, physical performance, muscle CSA/thickness, and muscle strength. The current literature suggests strong evidence (GRADE A) supporting increases in tendon CSA and tendon stiffness when collagen supplementation is paired with structured, appropriately loaded resistance training. The current literature suggests conflicting evidence (GRADE C) for physical performance outcomes, with some studies showing improvements in eccentric force production and neuromuscular function during stretch-shortening cycle tasks, and others showing no effect, suggesting that benefits may be task-specific. There is conflicting evidence (Grade C) for changes in muscle CSA, and strong evidence (GRADE A) against an effect on muscle strength beyond that achieved with resistance training alone. These findings build on the previous literature by identifying dose-dependent and tissue-specific effects of collagen, particularly in collagen-rich structures such as tendons, and by providing evidence-graded recommendations for its application in both athletic and clinical settings.

Across the included studies, collagen supplementation combined with resistance training generally resulted in greater increases in tendon CSA compared to placebo. Lee [28] observed no between-group differences despite using a similar intervention. The authors proposed that the absence of between-group differences in CSA was likely attributable to the low frequency of high-intensity resistance training implemented (consisting of one session per week), and the substantial habitual tendon loading associated with soccer participation, which may have already elicited near-maximal CSA adaptations in this population. Three studies [31,34,35] reported notable hypertrophy favoring collagen-supplemented groups, with CSA increases ranging from approximately 6% to 11%. These findings support the hypothesis that collagen supplementation may enhance tendon remodeling when paired with sufficient mechanical stimulus. To contextualize these findings, it is important to consider the biological mechanisms through which collagen supplementation and mechanical loading influence tendon hypertrophy. This adaptation is likely driven by the synergistic effects of repeated loading and increased collagen bioavailability, which together stimulate collagen synthesis [3,37]. Type I collagen-regulatory factors such as transforming growth factor β_1_ (TGF-β_1_); the tenocyte-specific transcription factor scleraxis (SCXA); and lysyl oxidase, the primary enzyme involved in collagen cross-linking, are upregulated in tenocytes following mechanical strain [3,4]. This stimulates extracellular matrix production and contributes to tendon hypertrophy and increased CSA. While mechanical loading (i.e., resistance training) initiates the cellular signaling required for tendon remodeling, the provision of exogenous hydrolyzed collagen or collagen peptides may further enhance this process by supplying the necessary amino acid precursors for new collagen synthesis. Ingesting collagen leads to a marked rise in circulating collagen-specific amino acids such as glycine, proline, hydroxyproline, and hydroxylysine, which peak approximately one-hour post-consumption [8,9]. Accordingly, timing collagen intake 60 min before the training stimulus may be critical to ensuring that these amino acids are bioavailable when mechanical loading occurs, thereby maximizing their incorporation into tendon tissue. While these biological mechanisms provide a plausible rationale for collagen supplementation, the included clinical trials primarily evaluated structural and performance outcomes rather than directly measuring molecular mechanisms of tendon remodeling. These findings indicate that while tendon hypertrophy can occur with training alone, collagen supplementation may further amplify this adaptation when implemented under appropriate physiological and programmatic conditions.

Four studies [28,31,34,35] investigated the effects of collagen supplementation combined with resistance training on tendon stiffness, with two of these studies [28,35] also evaluating Young’s modulus. Significant increases in tendon stiffness favoring collagen supplementation were reported by Lee ([28]; +15.4%) and Nulty ([35]; +56.4%), who administered a higher dosage of hydrolyzed collagen with vitamin C (30 g/day) alongside structured resistance training protocols. Conversely, Jerger [31,34], employing lower-dose supplementation (5 g/day collagen peptides), demonstrated increases in tendon stiffness within both collagen and placebo groups (approximately 16–35%), but without significant group–time interactions. Across the included studies, trials using higher collagen doses (15–30 g/day) tended to report larger increases in tendon stiffness compared with studies using lower doses (~5 g/day). It should be noted that these observations represent cross-trial comparisons rather than direct dose–response evidence, as none of the included randomized controlled trials directly compared multiple collagen dosages within the same experimental design. Consequently, these findings should be interpreted as hypothesis-generating rather than confirmatory.

Young’s modulus, reflecting tendon material properties independent of tendon size, increased significantly with collagen supplementation compared to placebo in the two higher-dose studies (Lee [28]: +14.2%; Nulty [35]: +46.2). Jerger [31,34] did not evaluate Young’s modulus, limiting direct comparisons at lower collagen dosages. Nevertheless, the consistent increases in Young’s modulus observed in higher-dosage studies further support the hypothesis that supplementation with hydrolyzed collagen positively influences intrinsic tendon tissue properties when combined with resistance training.

Increases in tendon stiffness and Young’s modulus represent structural and compositional adaptations, including augmented collagen fibril density, improved fibrillar alignment, and enhanced collagen cross-link formation [10,11,12]. Mechanical loading independently promotes these adaptations through stimulation of tenocyte activity and enzymatic processes, primarily involving lysyl oxidase-mediated collagen cross-linking [3,4,9]. A systematic review and meta-analysis revealed that increases in modulus, rather than tendon CSA, are the primary mediators of training-induced enhancements in tendon stiffness [18]. These adaptations are thought to result primarily from increased gene expression associated with anabolic responses to mechanical strain, which promotes collagen synthesis, turnover, and enhanced enzymatic cross-linking of collagen fibers [3,38]. Both tendon stiffness and Young’s modulus adaptations are highly dependent upon loading intensity. High-intensity resistance training (i.e., 70–90% 1 RM) leads to greater overload of the muscle–tendon unit and thus generates greater tendon strain, eliciting a stronger adaptive stimulus [10,39,40]. Supplementation with higher doses (15–30 g) of hydrolyzed collagen may further potentiate these remodeling processes by increasing the availability of collagen-specific amino acid precursors necessary for collagen synthesis. Recent findings from an RCT demonstrate that 30 g of hydrolyzed collagen with 50 mg of vitamin C consumed prior to resistance exercise significantly increases whole-body collagen synthesis compared to lower doses [41,42]. Consequently, the observed superior increases in tendon stiffness and Young’s modulus following high-dose collagen supplementation are likely attributable to augmented mechanical loading-induced collagen synthesis occurring in the presence of elevated serum concentrations of essential amino acids required for collagen formation [9,20,35,37,41]. The existing evidence thus strongly supports the efficacy of higher dose (approximately 15–30 g/day) hydrolyzed collagen supplementation in eliciting meaningful improvements in tendon stiffness and Young’s modulus when integrated with structured mechanical loading protocols. These structural adaptations are biomechanically meaningful, as increased tendon stiffness and CSA are associated with improved force transmission, stretch–shortening cycle efficiency, and greater tendon load tolerance; however, the extent to which collagen-induced structural changes translate to consistent improvements in functional or clinical outcomes warrants further investigation.

The most robust performance-related effects of collagen supplementation observed across studies were in eccentric rate of force development (eccentric RFD) and deceleration impulse, as reported by Lis [33]. Although no between-group differences were found for gross explosive performance outcomes such as countermovement jump (CMJ) height or squat jump (SJ), the HC + C group maintained maximal isometric RFD at baseline levels (−2.13 ± 5.20%), while the placebo group exhibited a marked decline (−16.20 ± 4.00%). Statistically significant group x time interactions favored the treatment group for eccentric RFD (*p* = 0.04, eccentric deceleration RFD (*p* = 0.008) and deceleration impulse (*p* = 0.03). While these metrics are not yet widely established as standard indicators of stretch-shortening cycle (SSC) function, Lis et al. [33] suggests that they reflect the neuromuscular capacity to absorb and reverse momentum during the eccentric phase of explosive movement. Previous studies have demonstrated that improved eccentric mechanics during the countermovement phase can increase power output by enhancing pre-activation of the lower limb musculature and facilitating more efficient transition between eccentric and concentric actions [43,44]. In this context, the significant improvements observed in the collagen-supplemented group may reflect enhanced mechanical efficiency during SSC tasks. Additionally, while isometric RFD is highly sensitive to neuromuscular fatigue and muscle damage [45,46], Lis et al. [33] reported that only the treatment group recovered to baseline RFD levels by the final testing session, potentially indicating a collagen-mediated benefit for neuromuscular recovery under high training demands.

The lack of improvement in CMJ height, despite gains in eccentric metrics, highlights recognized dissociation between underlying SSC mechanics and overt performance outcomes [47]. Enhancements in eccentric braking and force absorption capacity may not immediately translate to greater vertical displacement, particularly under the influence of concurrent strength and power training [48]. Although a statistically significant increase in leg spring stiffness was not retained due to outlier removal, the observed trend in the HC + C group supports the theoretical model that stiffer tendons improve SSC efficiency by enabling faster force transmission and reducing the energetic cost of movement [2,19]. As further evidence supporting an eccentric-specific adaptation, Lis et al. [33] observed that improvements were present in CMJ performance but not in SJ metrics. This distinction is meaningful, as the CMJ incorporates a rapid eccentric loading phase that facilitates elastic energy storage and neuromechanical coupling, whereas SJ begins from a static position and lacks this eccentric component, thereby limiting the contribution of the stretch-shortening cycle. These findings suggest that collagen supplementation may not directly enhance maximal concentric power but rather optimize elastic energy return and neuromechanical readiness in reactive movements.

These conclusions are further informed by findings from Nulty [35], who reported significant improvements in performance outcomes, including isometric RTD, 10-RM, CMJ, and broad jump following high-intensity resistance training in both groups. However, no group x time interactions were observed, indicating that collagen supplementation did not enhance these performance gains beyond training alone [35]. This finding is notable given that the collagen group also demonstrated greater increases in tendon stiffness and Young’s modulus elsewhere in the study, yet these mechanical adaptations did not translate into superior improvements in jump performance or explosive strength. Nulty [35] suggest that high inter-individual variability in neuromuscular activation likely influenced by the participants’ lack of prior resistance training experience may have overshadowed any subtle ergogenic effects of collagen on early-phase force production [49].

Unlike Lis et al. [33], who evaluated eccentric-specific metrics such as deceleration impulse and eccentric RFD, Nulty [35] focused on general explosive strength outcomes that may be less sensitive to tendon-mediated enhancements. In summary, while both studies support the role of resistance training in improving neuromuscular performance, only Lis et al. [33] demonstrated a clear, performance-relevant advantage attributable to collagen supplementation. These findings suggest that the performance-enhancing effects of collagen supplementation may not manifest uniformly across all task types, but rather are most likely to emerge during movements that heavily engage the stretch-shortening cycle and require rapid eccentric–concentric transitions, where tendon behavior plays a more active role in force transmission and elastic energy return.

The effects of collagen supplementation combined with resistance training on muscle CSA, thickness, and strength varied across studies. Two studies [32,34] reported statistically significant improvements in muscle hypertrophy favoring collagen supplementation, particularly in the gastrocnemius and quadriceps muscle groups. Conversely, two other studies [30,35] found no additional hypertrophic benefits of collagen supplementation beyond resistance training alone. All included studies consistently demonstrate significant improvements in muscle strength following resistance training alone; however, no additional strength benefits from collagen supplementation were observed. This consistent finding across multiple strength assessments suggests that collagen has a minimal direct effect on muscle contractile properties.

The divergent findings in muscle hypertrophy outcomes observed across the included studies may be explained, in part, by indirect mechanisms associated with collagen supplementation. While collagen peptides possess lower anabolic potential compared to leucine-rich proteins such as whey, due to their limited leucine content [6,50,51] they may still promote muscle adaptation through alternative pathways. Specifically, collagen peptides are rich in glycine, proline, and arginine, amino acids that contribute to extracellular matrix remodeling and connective tissue integrity [8,9,52]. Recent evidence further supports a potential role for collagen peptides in modulating myocellular signaling. Collagen supplementation may support connective tissue remodeling; however, unlike high-quality protein sources, it does not meaningfully stimulate canonical anabolic signaling pathways associated with skeletal muscle hypertrophy, likely due to its low essential amino acid and leucine content [51,52]. While some studies report improvements in fat-free mass with collagen supplementation [32,34], the underlying molecular mechanisms remain unclear. Although these pathways are traditionally associated with leucine-based signaling, these findings suggest that collagen peptides may exert modest anabolic effects when paired with resistance training.

The existing evidence does not support collagen supplementation to augment muscle strength beyond that achieved through resistance training alone, but it may modestly enhance muscle hypertrophy in specific contexts. These effects are likely mediated through a combination of improved connective tissue quality and secondary activation of anabolic signaling pathways, particularly when collagen is consumed in sufficient doses and timed appropriately with resistance exercise. Additionally, the reporting and control of total daily protein intake varied across the included trials, and in several studies, habitual dietary protein intake was not explicitly standardized. Because collagen supplementation may influence total protein intake, this factor may represent a potential confounder when interpreting muscle-related outcomes. It should also be noted that the majority of participants across the included studies were male, thus limiting the generalizability of these findings to female populations and highlighting the need for future research examining the effects of collagen supplementation on tendon adaptations in women.

### Limitations

Several limitations must be acknowledged when interpreting the results of this systematic review. While all included studies utilized randomized controlled designs, the methodological quality varied, with some lacking detailed reporting on allocation concealment, blinding, and power calculations. Sample sizes were generally small, limiting generalizability and statistical power. There was also considerable heterogeneity in collagen supplementation protocols, including differences in collagen type, dosage (5–30 g/day), timing (pre- vs. post-exercise), and whether vitamin C was co-ingested. This variability complicates the identification of an optimal supplementation strategy.

Additional limitations include inconsistent resistance training protocols across studies, with variations in frequency, intensity, and supervision potentially influencing outcomes. Additionally, because all included studies combined collagen supplementation with structured exercise interventions, it remains difficult to fully distinguish the independent contribution of supplementation from training-induced adaptations. Outcome measures were also not standardized, as imaging techniques, anatomical landmarks, and performance tests varied widely, reducing comparability across trials. In instances where paired data were unavailable, the variability of change scores was estimated using a recognized and widely applied approximation that assumes independence between pre- and post-intervention measurements. Although this approach does not account for the correlation inherent in repeated measures, it is considered a pragmatic and methodologically acceptable solution in the absence of the necessary data, and it facilitates the derivation of interpretable interval-based estimates of variability. Most participants were healthy, young adult males, with limited representation of older adults, females, or clinical populations. Participant populations also varied across studies and included both trained athletes and recreationally active or untrained individuals, which may influence baseline tendon properties and adaptive responses to collagen supplementation. Additionally, intervention durations were relatively short (3–15 weeks), which may not have been sufficient to capture long-term adaptations. Furthermore, the possibility of publication bias cannot be excluded, as studies reporting positive findings may be more likely to be published than those reporting null results. These limitations underscore the need for future trials to employ standardized training and testing protocols, recruit more diverse populations, and incorporate longer follow-up periods to better elucidate the role of collagen supplementation in musculoskeletal adaptation.

Several limitations related to the review process should also be acknowledged. Although multiple databases were searched, grey literature and trial registries were not systematically screened, which may introduce publication bias. Additionally, no quantitative meta-analysis or formal assessment of small-study effects was performed due to methodological heterogeneity across interventions and outcome measures. Consequently, the potential influence of publication bias or small-study effects within the current evidence base cannot be fully excluded. This review was limited to published studies available in English, which may have excluded relevant data.

## 5. Conclusions

The findings of this systematic review provide a provisional evidence-informed framework for collagen supplementation strategies in athletic and clinical settings, summarizing intervention characteristics commonly used across the included randomized controlled trials rather than establishing definitive clinical guidelines with strong evidence (GRADE A) based on the predominance of randomized controlled trials supporting increases in tendon cross-sectional area and tendon stiffness, strong evidence (GRADE A) against an effect on muscle strength, and conflicting evidence (GRADE C) for muscle cross-sectional area and physical performance. This framework reflects intervention characteristics reported across the included trials and should not be interpreted as a prescriptive clinical guideline. First, supplementation should be paired with structured, high-intensity resistance training (≥70% 1 RM), as mechanical loading is the primary stimulus driving tendon and muscle adaptations. Available evidence suggests that higher doses of hydrolyzed collagen (15–30 g/day) may be more effective than lower doses, particularly for enhancing tendon stiffness and Young’s modulus. Ingesting collagen approximately 60 min prior to training appears to be the most physiologically advantageous timing, as this coincides with peak serum concentrations of collagen-specific amino acids (e.g., glycine, proline, and hydroxyproline), thereby enhancing their availability during the collagen synthesis window initiated by mechanical strain. Supplementation can be limited to training days only, as the mechanical stimulus is necessary for collagen incorporation into target tissues. Collagen should be co-ingested with vitamin C (≥50 mg) to facilitate extracellular cross-linking of collagen fibrils [7,8]. Future studies should clarify the dose–response relationship across varied training loads, determine the minimal effective dose, and examine long-term adaptations in different populations, including aging adults and athletes undergoing high tendon stress.

## Figures and Tables

**Table 1:** PICO question and study design inclusion and exclusion criteria.

Question Component	Inclusion Criteria	Exclusion Criteria
Population	• Studies involving human participants of any age, sex, or ethnicity. • Participants with healthy tendons or pathological tendons.	• Studies involving animal models or non-human subjects. • Studies focusing exclusively on populations with underlying comorbidities that may confound tendon health (e.g., severe systemic diseases).
Intervention	• Studies that investigate collagen supplementation (including hydrolyzed collagen, gelatin, or collagen peptides). • Studies that include proline or glycine as part of the supplementation regimen.	• Studies that do not specifically investigate collagen, gelatin, proline, or glycine supplementation.
Comparison	• Studies that include a control group receiving either a placebo or no treatment. • Studies comparing different types of collagen supplementation.	• Studies without a control group or appropriate comparison group.
Outcome	• Studies reporting on tendon health outcomes, including but not limited to: ○ Tendon structure; ○ Tendon mechanical properties; ○ Muscle performance; ○ Physical performance; ○ Pain levels associated with tendon injuries.	• Studies that do not report tendon health outcomes.
Study Design	• RCTs, cohort studies, and case–control studies. • Hand-selected articles that come from the review of references of the included studies.	• Non-peer-reviewed studies, abstracts, conference proceedings, or unpublished data. • Reviews, meta-analyses, or opinion pieces. • Studies without accessible full text.

Note: RCTs, randomized control trials.

**Table 2:** Level of evidence modified from the Oxford Center of Evidence Based Medicine (OCEBM).

Level of Evidence	Study Characteristics
I	Evidence obtained from high-quality randomized controlled trials, prospective studies, or diagnostic studies.
II	Evidence obtained from lesser quality randomized control trials, prospective studies, or diagnostic studies (e.g., improper randomization, no blinding, and <80% follow-up).
III	Case–control studies or retrospective studies.
IV	Case series.
V	Expert opinion.

**Table 3:** Grading of Recommendations Assessment, Development, and Evaluation (GRADE) Working Group Criteria.

Grade of Recommendation		Strength of Evidence
A	Strong	A preponderance of level I and/or level II studies supports the recommendation. Must include ≥1 level I study.
B	Moderate	A single high-quality randomized controlled trial or a preponderance of level II studies supports the recommendation.
C	Weak	A single level II study or a preponderance of level III and level IV studies including statements of consensus by content experts supports the recommendation.
D	Conflicting	Higher-quality studies conducted on this topic disagree with respect to their conclusions. The recommendation is based on these conflicting studies.
E	Theoretical/Foundational	A preponderance of evidence from animal or cadaver studies, from conceptual models/principles, or from basic sciences/bench research supports this conclusion.
F	Expert Opinion	Best practice based on the clinical experience of the guideline-development team.

Note: Adapted from [26].

**Table 4:** Characteristics of included studies.

Primary Author (Year)	Participants				Study Design	Methods	Primary Outcomes:	Primary Results:
	N =	Male: Female	Age (Years)	Other Characteristics				
Lee [28]	11	0:11	25.7 ± 4.2	Professional female soccer athletes	RCT	RT + HC w/VC	• PT mechanical properties • PT material properties	• ↑ Increased Stiffness and YM • No effect on PT CSA
Nunez-Lisboa [29]	9	9:0	32.5 ± 4.1	Male triathletes	RCT	PS + HC	• $K_{vert}$ • Spatiotemporal parameters	• $\;\mathrm{No}\;\mathrm{effect}\;\mathrm{on}\;K_{vert}$ or spatiotemporal parameters
Kirmse [30]	57	57:0	24.0 ± 3	Moderately trained males	RCT	RT + CP	• Body Composition • Strength • Muscle fCSA	• No effect on Muscle strength or fCSA
Jerger [31]	31	31:0	28.6 ± 5.1	Healthy males with low/moderate physical activity (<120 min per week)	RCT	RT + CP	• PT CSA and stiffness • Maximal knee extension strength	• ↑Increased PT CSA • No effect on PT Stiffness, knee extension strength
Balshaw [32]	39	39:0	CP: 27.0 ± 5.0 PLA: 24.4 ± 3.2	Healthy males with low/moderate level of recreational physical activity	RCT	RT + CP	• Functional, structural, and contractile adaptations of skeletal muscle	• ↑IncreasedMuscle volume, twitch peak torque, architectural remodeling • No effect on muscle strength
Lis [33]	50	50:0	18–25	Healthy male athletes participating in football, rugby, or ROTC	RCT	PWT + HC w/VC	• RFD	• ↑ Increased change in CM jumps eccentric deceleration impulse, eccentric deceleration RFD, recovery of RTD • No effect on maximal isometric squat force
Jerger [34]	40	40:0	26.3 ± 4.0	Healthy males with no recent history (>12 months) of RT or not involved in any systematic training (>60 min per week)	RCT	RT + CP	• AT CSA • AT stiffness • Muscular strength • Muscle thickness	• ↑Increased Tendon CSA, Muscle thickness • No effect on tendon stiffness, Muscle Strength
Nulty [35]	20	20:0	47 ± 5	Healthy males with >120 min moderate activity and/or sport training per week but naïve to LE resistance exercise	RCT	RT + HC w/VC	• PT CSA • PT Stiffness • Muscle strength • Muscle thickness • pRTD	• ↑Increased PT CSA, Youngs’ modulus, stiffness • No effect on strength, pRTD, and muscle thickness

Note: AT, Achilles tendon; CM, counter movement; CP, collagen peptide; fCSA; fiber cross-sectional area; HC, hydrolyzed collagen; $K_{vert}$, vertical stiffness; LE, lower extremity; min, minutes; pRTD, peak rate of torque development; PT, patellar tendon; PS, plyometric-specific; PWT, power training; RFD, rate of force development; RT, resistance training; VC, vitamin C; YM, Young’s modulus.

**Table 5:** Summary of risk of bias assessment for randomized control trials.

PEDro Criteria	Lee [28]	Nunez-Lisboa [29]	Kirmse [30]	Jerger [31]	Balshaw [32]	Lis [33]	Jerger [34]	Nulty [35]
1	√	√	√	√	√	√	√	√
2	√	√	√	√	√	√	√	√
3	**×**	**×**	√	√	√	**×**	**×**	√
4	√	√	√	√	√	√	√	√
5	√	√	√	√	√	√	√	√
6	**×**	**×**	**×**	**×**	**×**	**×**	**×**	**×**
7	√	**×**	**×**	**×**	**×**	**×**	**×**	√
8	√	√	√	√	√	√	√	√
9	**×**	**×**	**×**	**×**	**×**	**×**	**×**	**×**
10	√	√	√	√	√	√	√	√
11	√	√	√	√	√	√	√	√
Total	8	7	8	8	8	7	7	9
Interpretation	Good	Good	Good	Good	Good	Good	Good	Excellent

Note: Scores of ≤3 indicate poor quality; 4–5, fair quality; 6–8, good quality; and 9–10, excellent quality. Background colors are used to visually represent scoring outcomes across PEDro criteria (green = criterion met; red = criterion not met).

**Figure A1:**
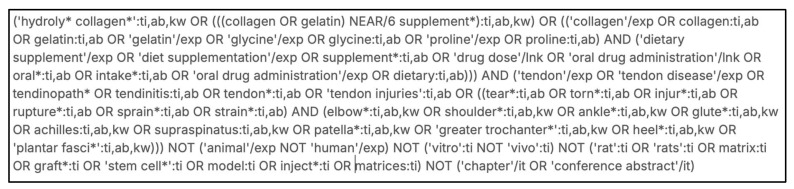
EMBASE search strategy.

## Data Availability

The data supporting the findings of this study are derived from publicly available randomized controlled trials cited within the manuscript. Extracted data and supporting materials are available from the corresponding author upon reasonable request. No additional analytic code was generated for this review.

## Appendix B

**Table A1:** Intervention prescription variables.

Primary Author, Year	Intervention Prescription Variables					
	Duration: Weeks	Training Frequency: Sessions Per/Week	Training Type	Ingestion Type	Ingestion Frequency	Ingestion Timing
Lee [28]	10	4 × RT + 1 × match	• EL UE RT • EL LE RT • EL LE plyometric exercises • Pitch-based sessions	30 g HC + 500 mg VC	3× per week: on days with training sessions	Before each training session
Nunez-Lisboa [29]	4	3 × PS	• Intervention-specific • PS: 4 × 100 m sprints post-supplementation • General triathlon training: • Collagen group: 16.6 ± 2.1 h/week • Control group: 17.3 ± 1.4 h/week	15 g HC (Great lakes gelatin,12 g of collagen hydrolysate, 36 mg of sodium)	3× per week: on Plyometric Stimulus Days	60 min before each training session
Kirmse [30]	12	3 × RT	• Whole-body RT • Squats, bench press, deadlift, bent-over-row, and knee extension • Each exercise: 1 set of 10 at 50% 1 RM, followed by 3 sets of 10 repetitions with 70% (weeks 4–12) 1 RM. Rest: 2 min	15 g CP (Bodybalance provided by Gelita AG)	Daily Ingestion	Training days: Immediately after training Non-training days: 24 h after previous ingestion
Jerger [31]	14	3 × RT	• Whole-body RT • Leg press, knee extension, and calf raise (load progressed every four weeks, from 70% to 85% 1 RM) • Latissimus pull + bench press added to increase compliance, performed after LE protocol	5 g CP (Tendoforte, provided by Gelita AG)	Daily Ingestion	Training days: Half ingested 30 min before training, half immediately after training Non-training days: 24 h after previous ingestion
Balshaw [32]	15	3 × RT	• LE RT • Unilateral knee extension, bilateral knee flexion, and leg press • 2–4 sets per exercise (4 sets for each exercise by week 7) with undulating periodization between ~12 RM and ~6 RM	15 g CP (10 g of BodyBalance and 5 g of Tendoforte, Gelita AG)	Daily Ingestion	Training days: Immediately after training Non-training days: Mid-afternoon
Lis [33]	3	3 × PWT	• PWT • Progressive loading, including vertical drop jumps, vertical box jumps, and body weight loaded ballistic squats • Rugby and ROTC participants: ○ First 3 days: 2 × 10 reps, progressed to 3 × 10, reached 5 × 10 reps in final week • Football players: ○ Progressive PWT program: ballistic squats, plyometrics, and speed work, without the same progression	20 g HC + 50 mg VC	Daily Ingestion	Training days: ∼60 min before training Non-Training Days: Ingestion with breakfast
Jerger [34]	14	3 × LE RT	• LE RT: • Seated and standing calf • Progressively increasing load from 70% to 85% 1 RM over 14 weeks, 3 sets per exercise, with reps decreasing from 12 to 6	5 g CP (Tendoforte, provided by Gelita AG)	Daily Ingestion	Training days: Within one hour after training Non-training days: 24 h after previous ingestion
Nulty [35]	12	2 × LE RT	• LE RT: • Barbell back squats, dumbbell Romanian deadlifts, trap-bar deadlifts and dumbbell goblet squats. • Starting: 4 sets of 8–10 reps at 90% 10-RM for 6 weeks, then progressed to 6–8 reps for the remaining 6 weeks; loads increased weekly based on performance	30 g HC (collagen protein, MyProtein) +50 mg VC	2× per week: on training days	Immediately post-training (5 min)

Note: EL, externally loaded; h, hours; HC, hydrolyzed collagen; LE, lower extremity; min, minutes; PS, plyometric stimulus; PWT, power training; reps, repetitions; RM, repetition maximum; RT, resistance training; UE, upper extremity; VC, vitamin C.
